# Transcriptome Profiling of Induced Sputum Identified Upregulated TNF-α/NF-κB Signalling and Downregulated Mitochondrial Respiratory Chain Function in Post-Infectious Bronchiolitis Obliterans

**DOI:** 10.3390/biom16050745

**Published:** 2026-05-19

**Authors:** Silvija P. Jerkic, Karen Naegele, Lucia Gronau, Annika Detring, Jordis Trischler, Katharina Blümchen, Björn Rotter, Mohammed Alkhatib, Margarete Mijatovic, Andreas Weigert, Andreas G. Chiocchetti, Stefan Zielen, Ralf Schubert

**Affiliations:** 1 Department of Pediatrics, Division of Pneumology, Allergology, Infectious Diseases and Gastroenterology, Goethe University Frankfurt, Theodor-Stern-Kai 7, 60590 Frankfurt am Main, Germany; karen.naegele@t-online.de (K.N.); 95lucy@googlemail.com (L.G.); annika.detring@icloud.com (A.D.); trischler@med.uni-frankfurt.de (J.T.); bluemchen@med.uni-frankfurt.de (K.B.); r.schubert@med.uni-frankfurt.de (R.S.); 2 GenXPro GmbH, 60438 Frankfurt am Main, Germany; b.rotter@genxpro.de (B.R.); m.khatib@genxpro.de (M.A.); 3 Institute of Biochemistry I, Faculty of Medicine, Goethe University Frankfurt, 60629 Frankfurt am Main, Germany; mijatovic@biochem.uni-frankfurt.de (M.M.); weigert@biochem.uni-frankfurt.de (A.W.); 4 Department for Immunity of Inflammation, Medical Faculty Mannheim, Heidelberg University, 60590 Mannheim, Germany; 5 Department of Child and Adolescent Psychiatry, Psychosomatics and Psychotherapy, University Hospital Frankfurt, Goethe Universität, 60590 Frankfurt Am Main, Germany; andreas.chiocchetti@med.uni-frankfurt.de; 6 Respiratory Research Institute, Medaimun GmbH, 60596 Frankfurt am Main, Germany; s.zielen@medaimun.de

**Keywords:** post-infectious bronchiolitis obliterans, paediatric pulmonology, induced sputum, transcriptome profiling, mitochondrial respiratory chain function

## Abstract

Post-infectious bronchiolitis obliterans (PiBO) is a chronic lung disease that develops after severe lower respiratory infections and leads to persistent inflammation and fibrotic changes in the small airways. In the present study, gene expression analysis was used to identify differentially expressed genes (DEGs) in sputum cells derived from PiBO patients and compare them to healthy controls. Clinical history, lung function parameters, and induced sputum samples were collected from nine patients with PiBO and eight healthy controls. Multiplex immunohistochemistry (mIHC) as well as mRNA sequencing (MACE-Seq) were performed. Evaluation of the biological targets was done by KEGG pathway enrichment analysis. PiBO patients showed significantly reduced lung function parameters, an increased neutrophil count, and an altered macrophage profile in sputum. Transcriptome analysis revealed significant upregulation of the TNFα-dependent NFκB signalling pathway, as well as significant downregulation of the oxidative phosphorylation (OXPHOS). Linear regression analyses and mIHC indicated a shift in macrophage polarisation that may contribute to the dysregulated gene expression. Notably, expression of these DEGs significantly correlated with FEV_1_ lung function. These findings indicate a central role of macrophages in the immunopathology of PiBO and contribute to our understanding of the molecular mechanisms involved in the disease process.

## 1. Introduction

Post-infectious bronchiolitis obliterans (PiBO) is a chronic and irreversible lung disease that leads to obliteration of the small airways [1,2,3]. It occurs after severe pulmonary infections, causing damage to the lower respiratory tract, and is caused by various pathogens, such as adenovirus, influenza, measles, respiratory syncytial virus, and mycoplasma pneumonia. The inflammatory reaction progresses into fibrosis of the small airways and ultimately leads to progressive narrowing of the lumen [4]. Currently, there is no clear understanding of the mechanisms underlying the development of PiBO.

Analyses of induced sputum and bronchoalveolar lavage fluid (BALF) from patients with PiBO show a persistent neutrophilic inflammatory response, which is associated with permanently elevated concentrations of inflammatory cells and proinflammatory mediators, such as IL-1ß, IL-6, IL-8, MMP-9, and MPO [5,6,7].

A study conducted between 2002 and 2019 on 21 children with PIBO confirmed persistent neutrophilic inflammation in sputum over the years [8]. In this study, sputum samples were taken regularly during periods without exacerbation, and patients showed a steady increase in neutrophilic inflammation of 2.12% per year. These findings indicate that neutrophilia in the sputum of patients with PiBO is more likely to be an expression of a chronic, non-subsiding inflammatory process in the airways rather than a persistent infection. Although the pathogenesis of PiBO is not yet fully understood, impaired or incomplete resolution of inflammation is considered to be a central mechanism in the course of the disease.

This thesis is supported by data on fatty acid profiles connected with bronchial inflammation in patients with PiBO. Jerkic et al. demonstrated a reduced concentration of n-3 docosahexaenoic acid (DHA) in the blood and sputum of PiBO patients [9]. DHA is an anti-inflammatory omega-3 fatty acid and the launch product for resolution-promoting mediators such as resolvins, which connect fatty acid mediators and lead to resolution of inflammation [10].

Duecker et al. identified four dysregulated miRNAs—miR-335-5p, miR-186-5p, miR-30b-5p, and miR-30c-5p—in the peripheral blood of an international PiBO cohort that modulate signalling pathways relevant for the regulation of inflammatory and fibrotic processes [11]. These miRNAs are functionally involved in signalling pathways that control the balance between inflammation, its resolution, and fibrotic remodelling, including cytokine–cytokine receptor interactions, TGF-β signalling, and the FoxO signalling pathway. Notably, expression of these miRNAs correlated significantly with patients’ lung function parameters such as FEV_1_.

To gain deeper insights into the molecular mechanisms underlying the failure to resolve persistent inflammation, gene expression analysis was performed to identify differentially expressed genes (DEGs) in sputum cells derived from patients with PiBO.

## 2. Methods

### 2.1. Patients

Patients with PiBO and healthy, age-matched control subjects were recruited from the Department of Pediatrics at University Medicine Frankfurt, Goethe University, and clinical data, such as clinical history, lung function parameters, and induced sputum samples for cell differentiation and mRNA analysis, were collected from nine patients with PiBO (age: 16.7 ± 6.9 years; m/f: 7/2) and eight controls (age: 19.9 ± 7.9 years; m/f: 3/5) (Table 1). The diagnosis of PiBO was based on clinical history, with acute onset following a severe respiratory infection, impaired pulmonary function (PFT), and typical high-resolution computed tomography (HRCT) changes, including mosaic patterns, hyperinflation, and thickening of the bronchial walls [1]. Pathogens were identified in five patients (two with adenovirus, three with mycoplasma). Clinical symptoms included tachypnoea, cough, wheezing, exercise intolerance, and hypoxemia. In addition, there was evidence of impaired lung function according to the criteria of the National Institute of Health Clinical Center (NIH-CC).

This study was approved by the Ethics Committee of Goethe University Frankfurt (numbers 116/16 and 354/18) and complied with the ethical principles of the Declaration of Helsinki. Written consent was obtained from all patients and/or their legal guardians. All participants underwent pulmonary function testing and the collection, processing, and storage of induced sputum for further analysis.

### 2.2. Lung Function Tests

Baseline lung function was determined by body plethysmography (VIASYS Healthcare GmbH; Höchberg, Germany). The following parameters were recorded: FVC, FEV_1_, FEV_1_/FVC, and FEF75. Reversibility was defined as positive if a change of ≥12% and 200 mL was observed after bronchodilation.

### 2.3. Induced Sputum Collection and Processing

First, the study participants inhaled salbutamol and then hypertonic saline in increasing concentrations of 3%, 4%, and 5% NaCl for 7 min each. During this procedure, the participants rinsed and cleaned their noses to reduce the proportion of squamous epithelial cells in the samples. The sputum was processed within one hour of collection. The selected sputum plugs (with as little saliva as possible) were placed in a pre-weighed Eppendorf tube and treated with four times the weight/volume of 0.1% dithiothreitol (DTT). Then, twice the weight/volume of phosphate-buffered saline (PBS) was added. Cells were differentiated microscopically after May–Grünwald–Giemsa staining. At least 400 inflammatory cells were counted for each sample. The percentages of neutrophils, lymphocytes, eosinophils, and macrophages were determined using the method described [11]. The remaining cells were collected in mRNA buffer and stored at −80 °C for mRNA analysis.

### 2.4. RNA Extraction and Preparation and Sequencing of MACE Libraries

RNA was isolated from samples using the miRNeasy mini kit according to the manufacturer’s instructions (Qiagen, Hilden, Germany). Cells were lysed in QIAzol lysis reagent, and chloroform was added to perform a two-phase extraction. After centrifugation, the RNA in the aqueous phase was precipitated with ethanol and washed with the provided buffers from the kit using spin columns. Finally, it was eluted in 30 μL RNase-free water, and the concentration was determined using a NanoDrop ND-1000 spectrophotometer (Thermo Scientific, Dreieich, Germany). RNA integrity (RIN) was assessed using the Agilent RNA 6000 Nano Kit and the Agilent 2100 Bioanalyzer (Agilent Technologies, Santa Carla, CA, USA).

MACE-seq libraries were prepared by GenXPro (Frankfurt, Germany) as previously described using the MACE-Kit V2 according to the manufacturer’s instructions [12]. In brief, samples were treated with DNase I, and 200 ng of fragmented total RNA underwent reverse transcription using barcoded oligo(dT) primer containing TrueQuant unique molecular identifiers (GenXPro), followed by template switching. Polymerase chain reaction amplified libraries were purified by solid-phase reversible immobilisation beads, and subsequent sequencing was performed using the NextSeq 500 platform (Illumina), followed by TrueQuant (unique molecular identifiers) polymerase-chain-reaction bias elimination.

### 2.5. Cell Lines and Cell Stimulation

The human alveolar epithelial cell line A549, the human promyelocyte cell line HL60, and the monocytic cell line THP 1 were cultured as described by Gronau et al. and stimulated with a cytokine mixture (CM) consisting of 50 U/mL IL 1β, 400 U/mL IFN γ, and 20 ng/mL TNF α (PeproTech, Hamburg, Germany) [13]. The THP 1 cells were previously differentiated into M1 macrophages. For this purpose, priming was first performed with 5 ng/mL phorbol 12-myristate 13-acetate (PMA; Sigma Aldrich, Taufkirchen, Germany), followed by a 24-h resting phase in culture medium and a three-day polarisation with LPS 100 ng/mL.

### 2.6. Quantitative PCR (qPCR)

mRNA was isolated using the miRNeasy Mini Kit (Qiagen, Hilden, Germany) according to the manufacturer’s instructions. Cell culture samples were lysed in QIAzol lysis buffer (Qiagen), followed by the addition of chloroform. After centrifugation, the upper aqueous phase was removed, mixed with 100% ethanol, and applied to RNeasy Mini Spin Columns. The columns were washed, and the RNA was then eluted in RNase-free water. The concentration and purity of the RNA were determined using a NanoDrop ND-1000 spectrophotometer (NanoDrop Technologies, Wilmington, DE, USA). The purified RNA was stored at −80 °C until further use.

Total RNA was transcribed into cDNA using the QuantiTect™ Reverse Transcription Kit (Qiagen, Hilden, Germany) according to the manufacturer’s instructions. Prior to transcription, genomic DNA was removed by treatment with gDNA Wipeout Buffer. The thermocycler reactions were performed according to the kit protocol.

For qPCR, 2.5 ng/µL cDNA per sample was used with the TaqMan™ Fast Advanced Master Mix and specific TaqMan™ primers (Thermo Fisher Scientific, Dreieich, Germany). The reactions were performed on a QuantStudio 3 system (Thermo Fisher Scientific). GAPDH served as an endogenous reference, and relative gene expression was calculated using the 2^−ΔΔCt^ method.

### 2.7. Multiplex Immunohistochemistry

Sputum slides were processed using the Opal 7-Color Automation IHC Kit (Akoya BioSciences, Menlo Park, CA, USA) on a BOND-RX Multiplex IHC autostainer (Leica, Wetzlar, Germany). The primary antibodies used were anti-CD15 mAb (GA062, DAKO), anti-MPO mAb (A039829-2, DAKO), anti-CD68 mAb (M087601-2, DAKO), anti-MHCII mAb (68258S, Cell Signalling Technology, Boston, MA, USA), anti-CD163 mAb (ab182422, Abcam), and anti-CD206 mAb (91992S, Cell Signalling Technology). Cell nuclei were counterstained with 4′,6-diamidino-2-phenylindole (DAPI), and the preparations were then fixed with Fluoromount-G [14].

To optimise epitope retrieval and stability conditions, various antigen retrieval parameters, including pH and the order in the multiplex protocol, were systematically tested. Images were captured using the PhenoImager HT (Akoya Biosciences, Marlborough, MA, USA).

Cellular phenotypes were identified and classified using a trained machine learning algorithm in the InForm software (Akoya Biosciences, Marlborough, MA, USA). All images were analysed in batch mode, and the mean fluorescence intensity of each antibody–fluorophore pair, the assigned phenotypes, the Cartesian cell coordinates, and their relative frequencies were exported.

### 2.8. Bioinformatics and Statistical Analysis

Sequencing data from MACE-seq were uploaded and analysed on the GenXPro bioinformatics tools platform (https://tools.genxpro.net/). Raw Sequencing reads were adapter-trimmed, quality-trimmed and cleaned from library preparation artefacts using cutadapt (version 3.2). For UMI-containing libraries, PCR duplicates were removed by collapsing reads with identical UMI and insert sequences. Read quality was assessed using FastQC v0.11.9. Additional quality control metrics were generated using Samtools v1.12, and MultiQC v1.12 was used to summarise quality control results across samples.

Processed reads were mapped to the human GRCh38/hg38 reference genome using Bowtie2 v2.4.3. Gene-level annotation and quantification were performed separately using HTSeq v1.99.2 with the Ensembl GRCh38 release 110 gene annotation. Raw gene counts were used for differential expression analysis. Genes with low expression were removed before statistical testing. Only entries having at least a raw read count of 5 in at least 2 samples were retained for downstream analysis. Normalisation and differential expression testing were performed in R using DESeq2 v1.34.0. Raw counts were normalised using the median-of-ratios method implemented in DESeq2. Differentially expressed genes were defined as genes with an absolute log2 fold change ≥ 1 and a Benjamini–Hochberg false-discovery-rate adjusted *p*-value < 0.05.

Expression levels were additionally reported as transcripts per million (TPM) for descriptive expression summaries. TPM values were not used as input for DESeq2 differential expression testing. All differential gene expression analyses were adjusted for potential confounding by the percentage of granulocytes, macrophages, and lymphocytes, as well as by gender.

Baseline sample characteristics, including age, sex, sampling location, RNA quality, and sequencing read depth, were compared between groups using appropriate statistical tests according to variable type and distribution. No statistically significant differences were observed for these variables (all *p* > 0.1).

Protein–protein interaction enrichment analysis was performed using STRING (https://string-db.org/). A STRING-reported protein–protein interaction enrichment *p*-value < 0.05 was considered to be significant.

Statistical analyses of clinical and experimental variables were performed using Prism 10.0 (GraphPad Software 10.0, La Jolla, CA, USA). Group differences were assessed using Student’s *t*-test or analysis of variance for approximately normally distributed parametric data. When parametric assumptions were not met, non-parametric tests such as the Kruskal–Wallis test were used.

Linear regression analyses were performed to assess associations between selected differentially expressed genes and cell counts or FEV1. Given the small sample size, no formal multiple-testing correction was applied to these regression analyses, and the results were interpreted as exploratory and hypothesis-generating rather than confirmatory. We assessed assumptions primarily by graphical diagnostics. Residuals were visually inspected using Q-Q plots and residuals-versus-fitted plots, which did not indicate substantial deviations from normality or marked heteroskedasticity. Statistical significance was considered at *p* < 0.05.

## 3. Results

### 3.1. Lung Function Decline and Increased Airway Inflammation

A total of nine patients with PiBO and eight healthy control subjects were recruited from the children’s hospital in Frankfurt (Table 1). There were no significant differences between the two groups in terms of age or gender. Compared to the control group, PiBO patients had significantly reduced FVC, FEV_1_, FEV_1_/FVC, and FEF75. In addition, they displayed increased neutrophil and reduced macrophage counts in induced sputum.

### 3.2. Deregulated Gene Expression Implicated in Immune Regulation and Metabolic Pathways

**Figure 1:**
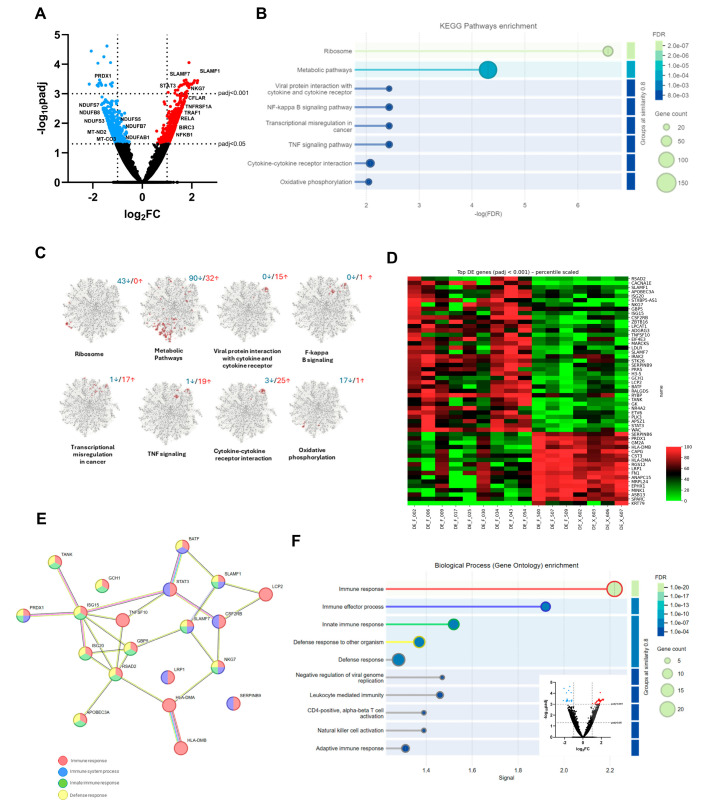
Deregulated genes in sputum of patients with post-infectious Bronchiolitis obliterans. (**A**) Volcano plot showing all differentially expressed genes (DEGs; *p*adj ≤ 0.05, base mean > 1), of which 629 were upregulated (red dots) and 565 were downregulated (blue dots) in the sputum of the PiBO patients compared to the controls. KEGG pathway analysis of (**B**) all DEGs with *p*adj ≤ 0.05 and (**C**) full gene interacting network was performed using STRING (v12.0, medium confidence score > 0.4). The red and blue numbers above the clusters indicate the number of genes that are upregulated or downregulated, respectively. (**D**) Heat map and (**E**) enriched terms of gene ontology (biological process) of the top DEGs (*p*adj < 0.001). (**F**) Network analysis shows hub genes that play a role in several of these processes.

An average of 6,729,639 reads per sputum sample was obtained from an average of 13,296 different genes. Analysis identified 1194 differentially expressed genes (DEGs; *p*adj. ≤ 0.05), of which 629 were upregulated and 565 were downregulated compared to controls (Figure 1A). KEGG pathway enrichment analysis revealed that these DEGs are involved in the following pathways: ribosome (hsa03010, fdr = 2.70 × 10^−7^), metabolic pathways (hsa01100, fdr = 4.97 × 10^−5^), viral protein interaction with cytokine and cytokine receptor (hsa04061, fdr = 0.0037), NF-kappa B signalling (hsa04064, fdr = 0.0037), transcriptional misregulation in cancer (hsa05202, fdr = 0.0037), TNF signalling (hsa04668, fdr = 0.0037), cytokine–cytokine receptor interaction (hsa04060, fdr = 0.0084), and oxidative phosphorylation (hsa00190, fdr = 0.0090) (Figure 1B). Interestingly, most genes involved in immune pathways were upregulated, while most genes associated with ribosome and metabolic pathways/oxidative phosphorylation were downregulated (Figure 1C). The heat map of the most significantly upregulated and downregulated DEGs with a *p*adj. < 0.001 revealed clear consistency between the patient and the control group (Figure 1D).

Gene ontology (biological process) enrichment analysis of these DEGs clearly demonstrated their involvement in immunoregulatory processes (Figure 1E). Top enriched terms included immune response (GO:0006955, fdr = 6.15 × 10^−20^); immune effector process (GO:0002252, fdr = 9.75 × 10^−8^); innate immune response (GO:0045087 fdr = 9.75 × 10^−8^); defence response to other organism (GO:0098542, fdr 9.30 × 10^−8^); defence response (GO:0006952, fdr = 1.13 × 10^−8^); negative regulation of viral genome replication (GO:0045071, fdr = 0.00028); leukocyte mediated immunity (GO:0002443, fdr = 5.54 × 10^−5^); CD4-positive, alpha-beta T cell activation (GO:0035710, fdr = 0.00041); natural killer cell activation (GO:0030101, fdr = 0.00042; and adaptive immune response (GO:0002250, fdr = 5.05 × 10^−5^). The network analysis (PPI enrichment *p* = 7.22 × 10^−12^) showed hub genes that play a role in several of these processes, such as *PRDX1*, *SLAMF1*, *SLAMF7*, and *NKG7* as well as *TANK*, *GCH1*, *ISG15*, *ISG20*, *APOBEC3A*, *RSAD2*, *GBP5*, and *BATF* (Figure 1F).

### 3.3. DEGs Are Involved in NF-κB Signalling and the Mitochondrial Respiratory Chain

**Figure 2:**
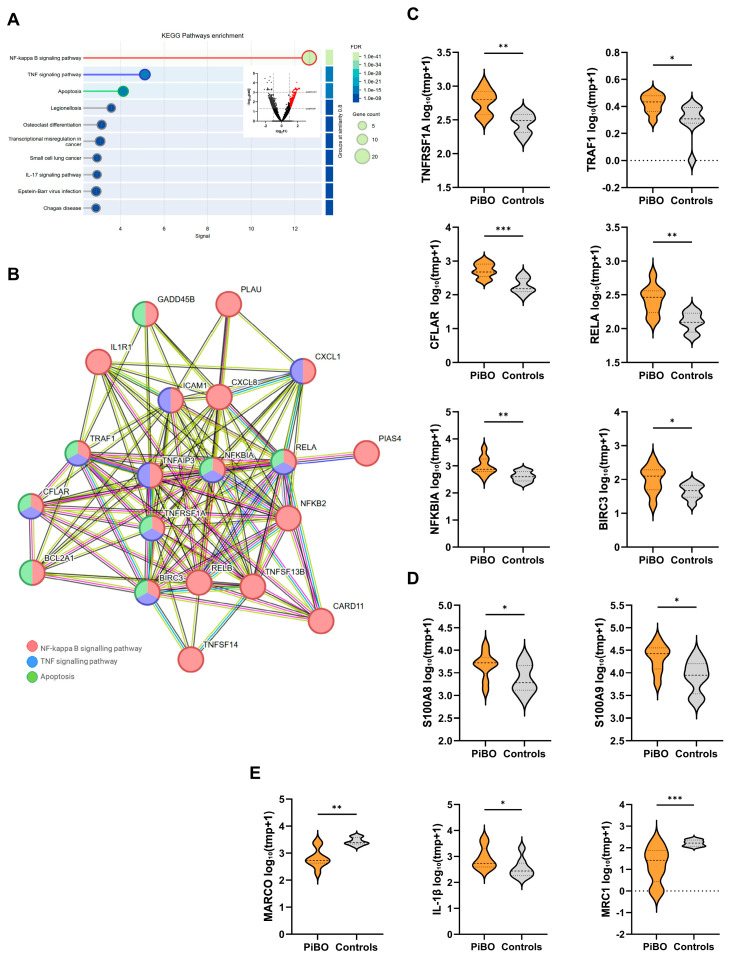
Signal pathways affected by the upregulated DEGs. (**A**) KEGG pathway enrichment of DEGs with *p*adj ≤ 0.05 and two-fold upregulation (base mean > 10). (**B**) Network analysis showing genes involved in NF-κB signalling, TNFα-signalling, and apoptosis. Violin plots depict normalised gene expression (log2(TPM + 1)) for (**C**) hub genes—*TNFRSF1*, *TRAF1*, *CFLAR*, *RELA*, *NFKBIA*, and *BIRC3*—that are involved in all three pathways, (**D**) genes specific for neurophils—*S100A8* and *S100A9*—and (**E**) genes specific for macrophages—*MARCO*, *Il-1β*, and *MRC1*. tpm, transcripts per million. * *p* < 0.05, ** *p* < 0.01, *** *p* < 0.001.

In the next step, we took a closer look at the upregulated and downregulated DEGs (*p*adj. *p* < 0.05) separately to identify key genes involved in the respective pathway. We identified 417 DEGs with *p*adj. < 0.05 and at least two-fold upregulation. KEGG pathway analysis of these genes revealed a strong enrichment of NF-κB signalling (hsa04064, fdr = 6.01 × 10^−41^) accompanied by TNFα-signalling (hsa04668, fdr = 7.68 × 10^−16^) and apoptosis (hsa 04215, fdr = 3.44 × 10^−13^) (Figure 2A,B). Six corresponding genes, *TNFRSF1*, *TRAF1*, *CFLAR*, *RELA*, *NFKBIA*, and *BIRC3*, were identified that are involved in all three pathways (Figure 2B,C). Comparison of the relative expression of the data showed significantly higher expression of *TNFRSF1* (PiBO 636 ± 109 tmp; controls 293 ± 33 tmp; *p* < 0.01), *TRAF1* (PiBO 63 ± 17 tmp; controls 15 ± 4 tmp; *p* < 0.05), *CFLAR* (PiBO 589 ± 111 tmp; controls 207 ± 40 tmp; *p* < 0.001), *RELA* (PiBO 296 ± 59 tmp; controls 131 ± 15 tmp; *p* < 0.01), *NFKBIA* (PiBO 1317 ± 445 tmp; controls 436 ± 66 tmp; *p* < 0.01), and *BIRC3* (PiBO 135 ± 39 tmp; controls 48 ± 8 tmp; *p* < 0.05) in the PiBO sputum samples compared to controls.

We also analysed the expression of genes specific for neutrophils and macrophages (Figure 2D). For neutrophil inflammation, we compared the expression of main calprotectin subunits and found both *S100A8* (PiBO 5617 ± 1331 tmp; controls 2907 ± 774 tmp; *p* < 0.05) and *S100 A9* (PiBO 26167 ± 5853 tmp; controls 10784 ± 2657 tmp; *p* < 0.05) to be significantly elevated in PiBO patients. To differentiate the macrophage populations, the expression of *MARCO* as a general marker for alveolar macrophages (AM), IL-1β as a marker for M1 macrophages, and *MRC1* as a marker for M2 macrophages were analysed (Figure 2E). *MARCO* was significantly downregulated in patients (PiBO 908 ± 300 tmp; controls 2826 ± 345 tmp; *p* < 0.01), which indicates a reduced number of total AMs. The expression of *IL-1β* (PiBO 936 ± 205 tmp; controls 453 ± 176 tmp; *p* < 0.05) was increased, whereas *MRC1* (PiBO 48 ± 21 tmp; controls 172 ± 20 tmp; *p* < 0.001) was decreased in PiBO patients compared to controls.

**Figure 3:**
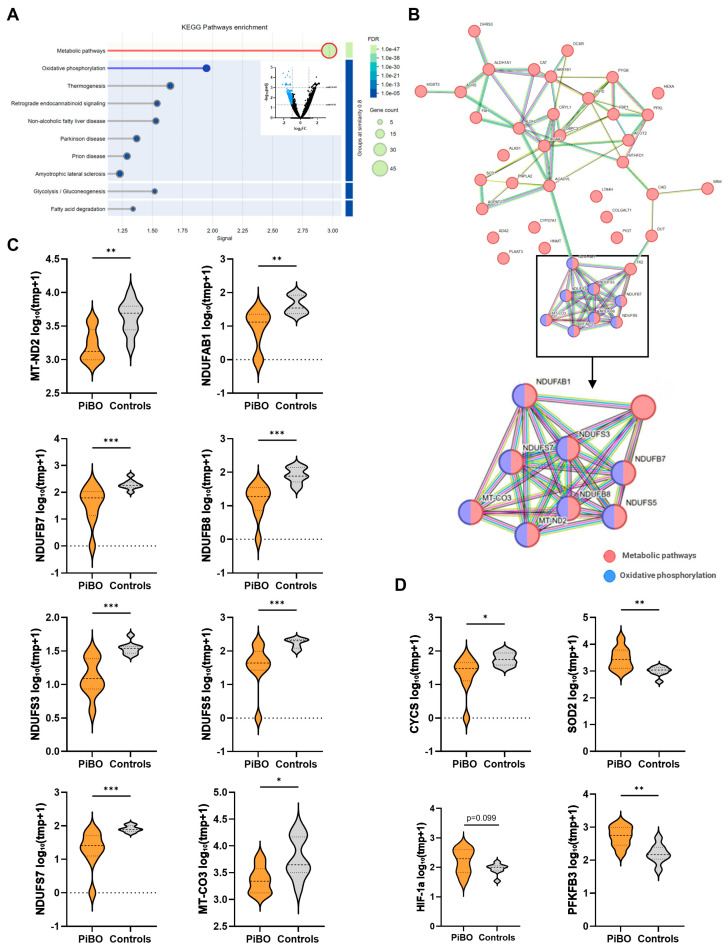
Signal pathways affected by the downregulated DEGs. (**A**) KEGG pathway enrichment of DEGs with *p*adj ≤ 0.05 and two-fold downregulation (base mean > 10). (**B**) Network analysis showing genes involved in metabolic pathways and oxidative phosphorylation. Violin plots depict normalised gene expression (log2(tpm + 1)) for (**C**) hub genes—*MT-CO3*, *MTND2*, *NDUFAB1*, *NDUFB7*, *NDUFB8*, *NDUFS3*, *NDUFS5*, and *NDUFS*—that are involved in both pathways and (**D**) genes associated with oxidative stress (*CYCS*, *SOD2*) and energy metabolism (*HIF-1α*, *PFKB3*). tpm, transcripts per million. * *p* < 0.05, ** *p* < 0.01, *** *p* < 0.001.

In addition to the upregulated genes, we also analysed DEGs that were significantly downregulated (*p*adj. < 0.05) with at least a two-fold decrease in expression. These 259 downregulated DEGs showed strong enrichment in metabolic pathways (hsa01100, fdr = 7.46 × 10^−47^) and oxidative phosphorylation (hsa00190, fdr = 8.19 × 10^−8^) (Figure 3A). Of the 43 DEGs involved in metabolic pathways, eight genes, *MT-CO3*, *MTND2*, *NDUFAB1*, *NDUFB7*, *NDUFB8*, *NDUFS3*, *NDUFS5*, and *NDUFS7*, were also involved in oxidative phosphorylation (Figure 3B,C). Analysis of relative expression revealed that the identified genes *MTND2* (PiBO 1873 ± 431 tmp; controls 4868 ± 887 tmp; *p* < 0.01), *NDUFAB1* (PiBO 13 ± 3 tmp; controls 49 ± 11 tmp; *p* < 0.01), *NDUFB7* (PiBO 68 ± 22 tmp; controls 210 ± 36 tmp; *p* < 0.001), *NDUFB8* (PiBO 19 ± 5 tmp; controls 90 ± 16 tmp; *p* < 0.001), *NDUFS3* (PiBO 14 ± 3 tmp; controls 35 ± 3 tmp; *p* < 0.001), *NDUFS5* (PiBO 63 ± 20 tmp; controls 178 ± 19 tmp; *p* < 0.001), *NDUFS7* (PiBO 30 ± 8 tmp; controls 83 ± 7 tmp; *p* < 0.001), and *MT-CO3* (PiBO 2711 ± 635 tmp; controls 8434 ± 2544 tmp; *p* < 0.01) were expressed at significantly lower levels in PiBO-derived sputum compared to controls (Figure 3C).

Due to the deregulated genes of the respiratory chain, we also observed the expression of genes associated with oxidative stress and energy metabolism (Figure 3D). Expression of cytochrome c (*CYCS*) was significantly downregulated (PiBO 30 ± 7 tmp; controls 62 ± 10 tmp; *p* < 0.05) in PiBO patients compared to controls, whereas expression of *SOD2* was significantly upregulated (PiBO 4448 ± 1668 tmp; controls 1044 ± 124 tmp; *p* < 0.01). Downregulation of the genes of the electron transport chain of complex I caused the cell to switch from oxidative phosphorylation of the mitochondria to a higher rate of glycolysis for ATP production. The data revealed increased expression of *PFKB3* (6-phosphofructo-2-kinase/fructose-2,6-biphosphatase 3) (PiBO 636 ± 149 tmp; controls 189 ± 47 tmp; *p* < 0.01) as a key enzyme in glycolysis and its regulator, hypoxia-inducible factor 1α (*HIF-1α*) (PiBO 232 ± 64 tmp; controls 94 ± 12 tmp; *p* = 0.099), in PiBO patients compared to controls.

### 3.4. Linear Regression Analysis of DEGs in Relation to Lung Function and the Number of Neutrophils and Macrophages in Sputum

Linear regression was used to model correlations between the expression of the DEGs and FEV1 lung function and neutrophil and macrophages count (Table 2, Figure S1). The Expression of *TNFRSF1*, *TRAF1*, *CFLAR*, and *RELA* as well as *MT-CO3*, *MTND2*, *NDUFAB1*, *NDUFB8*, *NDUFS3*, *NDUFS5*, and *NDUFS7* showed a significant correlation with FEV1, with the highest coefficients of determination for *RELA* (R^2^ = 0.49), *MTND2* (R^2^ = 0.52), and *NDUFS7* (R^2^ = 0.52). The expression of genes in the NF-κB signalling pathway was negatively correlated with FEV1, whereas the expression of OXPHOS genes was positively correlated. Gene expression of *RELA* as well as that of *MT-CO3*, *MTND2*, *NDUFAB1*, *NDUFB8*, and *NDUFS5* showed a significant correlation with neutrophil counts, whereby only *NDUFB8* (R^2^ = 0.52) achieved a coefficient of determination above R^2^ = 0.4. In contrast, the expression of almost all DEGs, except *TRAF1* and *BIRC3*, correlated significantly with macrophage counts, with *CFLAR* (R^2^ = 0.45), *MT-CO3* (R^2^ = 0.44), *MTND2* (R^2^ = 0.54), *NDUFAB1* (R^2^ = 0.60), *NDUFB8* (R^2^ = 0.75), *NDUFS3* (R^2^ = 0.40), *NDUFS5* (R^2^ = 0.73), and *NDUFS7* (R^2^ = 0.63) exhibiting relevant coefficients of determination. The number of macrophages correlated negatively with the expression of genes in the NF-κB signalling pathway and positively with OXPHOS gene expression, whereas the opposite pattern was observed for the number of neutrophils.

Overall, four out of six DEGs of the NF-κB signalling pathway and seven out of eight DEGs of the mitochondrial respiratory chain showed a significant correlation with FEV1 in lung function. The comparison between macrophages and neutrophils showed a significant correlation between four out of six DEGs of the NF-κB signalling pathway and between all DEGs of the mitochondrial respiratory chain and the macrophage counts, while the neutrophil counts only correlated significantly with one out of six DEGs of the NF-κB signalling pathway and five out of eight DEGs of the mitochondrial respiratory chain. Together with the higher R^2^-values, this suggests a stronger association between the DEGs and macrophages compared to neutrophil granulocytes.

### 3.5. A Shift in Macrophage Polarisation Appeared to Be Responsible for Dysregulated Inflammation and Oxidative Phosphorylation in Sputum

**Figure 4:**
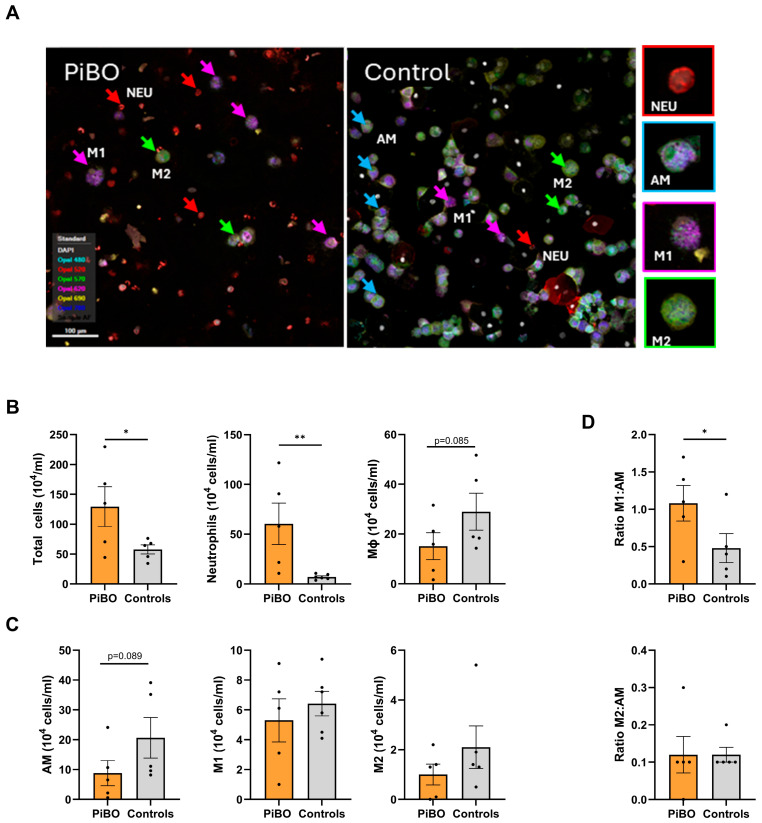
Shift in macrophage polarisation. Sputum slides were stained by multiplex immunohistochemistry (mIHC) using CD15 (cyan) and MPO (red) for neutrophils, CD68 for macrophages, MHCII (magenta) for M1 macrophages, and CD163 (yellow) and CD206 (green) for M2 macrophages. Cell nuclei were counterstained with DAPI (white). The cellular phenotypes of non-polarised alveolar macrophages (AM) and M1 and M2 macrophages were distinguished based on their intensity and parallel co-expression using a trained machine learning algorithm in the Form software. (**A**) Representative slides from a PiBO patient (left) and a healthy control (right). Scatter plot of absolute cell counts for (**B**) total cells, neutrophils, and macrophages and (**C**) macrophage phenotypes, calculated based on the relative frequency of mIHC data. (**D**) Ratio of M1 and M2 cells to AMs. * *p* < 0.05, ** *p* < 0.01.

We used mIHC staining to better differentiate between sputum cell populations, focusing on neutrophils, AMs, and M1 and M2 macrophages (Figure 4). AMs correspond to non-polarised cells, exhibiting a hybrid phenotype with strong CD68, moderate CD163/CD206, and moderate HLA-DR expression, allowing them to be distinguished by the strength and parallel co-expression of M1 polarised (HLA-DR very strong, CD163/CD206 weak) and M2 polarised (CD163/CD206 very strong, HLA-DR weak) macrophages.

As expected, mIHC staining confirmed the highly significant increase in the total cell count (PiBO 129 ± 33 10^4^/mL; controls 58 ± 8 10^4^/mL; *p* < 0.05) and numbers of neutrophil granulocytes (PiBO 60 ± 21 10^4^/mL; controls 7.1 ± 1.3 10^4^/mL; *p* < 0.01) in patient sputum (Figure 4A,B). In contrast, the number of macrophages (PiBO 15 ± 5.4 10^4^/mL; controls 29 ± 7.4 10^4^/mL; *p* = 0.085) tended to be reduced in the PiBO patients. The decrease in macrophage numbers appeared to be due to a reduction in the number of AMs (PiBO 8.8 ± 4.2 10^4^/mL; controls (21 ± 6.8 10^4^/mL; *p* = 0.089), while M1 and M2 numbers did not differ from the controls (Figure 4A,C). To analyse the relative polarisation of macrophages, we calculated the ratio of M1 and M2 cells to AMs (Figure 4D). We found a significant difference in the ratio of M1 cells (PiBO 1.07 ± 0.25 10^4^/mL; controls 0.48 ± 0.18 10^4^/mL; *p* < 0.05), but not M2 cells, compared to non-polarised AMs between patients and controls, indicating a shift in macrophage polarisation toward M1.

**Figure 5:**
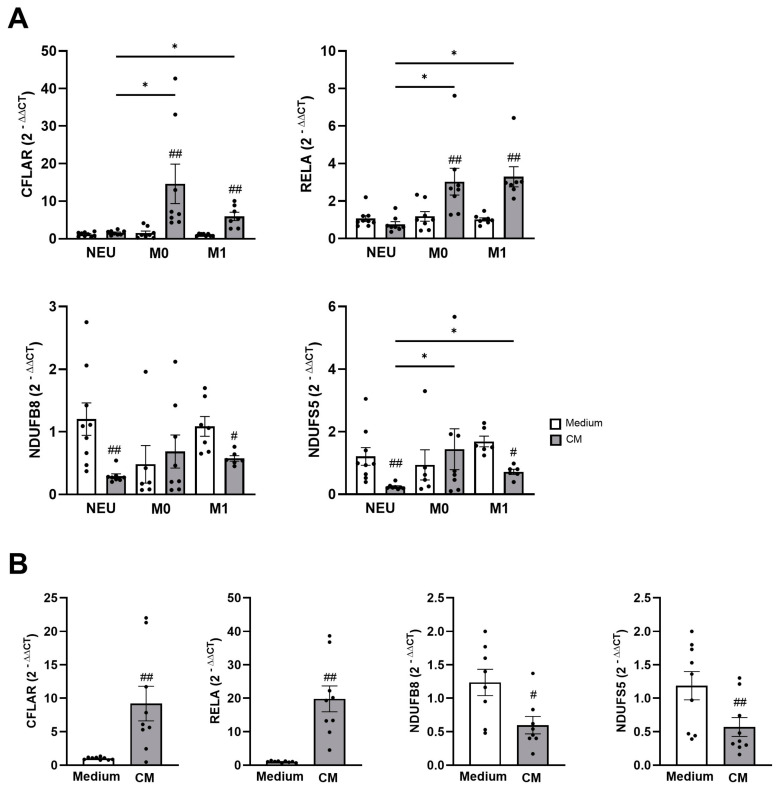
TNF-α/NF-κB pathway and oxidative phosphorylation in M1 macrophages and lung epithelial cells. The graphs show expression of *CFLAR* and *RELA* as well as *NDUFB8* and *NDUFS5* in (**A**) neutrophil precursor cells (HL60), M0 and M1 macrophages (THP-1), and (**B**) human alveolar epithelial cells (A549) after stimulation with a cytokine mixture (CM, IL-1β, IFN-γ, and TNF-α). Expression of the genes was performed by qPCR, and data are presented as 2^−ΔΔCT^. Significance between unstimulated and stimulated samples is indicated with # *p* < 0.05, ## *p* < 0.01 and between different cell types with * *p* < 0.05.

To investigate the extent to which the DEGs and signalling pathways were modulated in sputum neutrophils and macrophages during an inflammatory response, we stimulated neutrophil precursor cells (HL60) and M0 and M1 macrophages (THP-1) via the NF-κB signalling pathway. We selected *CFLAR* and *RELA* as well as *NDUFB8* and *NDUFS5* as the target genes, as these showed the strongest associations in the correlation analyses (Figure 5A). The stimulation led to a significant increase in the expression of *CFLAR* (M0 1.54 ± 0.52 to 14.61 ± 5.25 2^−ΔΔCT^, *p* < 0.01; M1 1.04 ± 0.11 to 5.95 ± 1.10 2^−ΔΔCT^, *p* < 0.01) and *RELA* (M0 1.18 ± 0.26 to 3.03 ± 0.71 2^−ΔΔCT^, *p* < 0.01; M1 1.02 ± 0.08 to 3.29 ± 0.54 2^−ΔΔCT^, *p* < 0.01) in M0 and M1 macrophages, respectively, but not in neutrophils. In contrast, stimulation resulted in a significant reduction in the expression of *NDUFB8* (NEU 1.20 ± 0.26 to 0.29 ± 0.04 2^−ΔΔCT^, *p* < 0.01; M1 1.09 ± 0.16 to 0.58 ± 0.05 2^−ΔΔCT^, *p* < 0.05) and *NDUFS5* (NEU 1.21 ± 0.28 to 0.24 ± 0.03 2^−ΔΔCT^, *p* < 0.01; M1 1.69 ± 0.17 to 0.70 ± 0.08 2^−ΔΔCT^, *p* < 0.05) in neutrophils and M1 macrophages, but not in resting M0 macrophages.

Given the strong FEV1 correlations with the expression of *CFLAR*, *RELA*, *NDUFB8*, and *NDUFS5*, we additionally analysed their modulation in the lung epithelial cell line A549 (Figure 5). Stimulation of A549 cells resulted in a significant increase in the expression of *CFLAR* (from 1.02 ± 0.07 to 9.19 ± 2.58 2^−ΔΔCT^, *p* < 0.01) and *RELA* (from 1.03 ± 0.08 to 19.78 ± 3.85 2^−ΔΔCT^, *p* < 0.01). Vice versa, stimulation led to a significant decrease in the expression of *NDUFB8* (from 1.24 ± 0.20 to 0.60 ± 0.13 2^−ΔΔCT^, *p* < 0.05) and *NDUFS5* (from 1.19 ± 0.21 to 0.57 ± 0.14 2^−ΔΔCT^, *p* < 0.01).

## 4. Discussion

The immunopathology of PiBO is not fully understood. Previous studies have repeatedly shown persistent neutrophilic inflammation in the small airways [5,6,8,9]. Further investigations have postulated that persistent neutrophilic inflammation leads to insufficient resolution of the inflammatory process and, thereafter, fibrosis and bronchiolar obstruction [5,7].

Clinically, patients with PiBO develop a significant flow limitation in the small airways, with a mixed obstructive and restrictive pattern in pulmonary function tests (PFTs) and air trapping in distal lung areas on HRCT. Clinical studies on PiBO patients have demonstrated a correlation between pulmonary obstruction on PFTs and elevated neutrophils in sputum and BAL [4,8,11].

In addition to the elevated neutrophils in sputum samples of patients with PiBO, further studies have revealed an increased number of proinflammatory cytokines, such as IL-1β, IL-6, IL-8, and TGF-β, which supports the initial findings and indicates persistent inflammation and fibrotic processes [5,6,11]. However, the exact mechanism of this persistent inflammation is not yet fully understood. This study used gene expression analysis to identify dysregulated genes involved in inflammatory signalling pathways in sputum cells from patients with PiBO.

Our analyses clearly showed dysregulated gene expression of immune regulation and the mitochondrial respiratory chain. A dysregulation of genes responsible for immune regulation was to be expected, as earlier studies in our centre indicated misdirected post-translational gene regulation of inflammation in PiBO [11], which was confirmed by this follow-up study. However, in this analysis, dysregulated gene expression of the mitochondrial respiratory chain was also shown.

Several studies on sputum gene expression in inflammatory lung diseases showed overexpression of immunoregulatory genes. Baines et al. found upregulated genes involved in IL-1 and TNF-α/NF-κB signalling pathway in patients with neutrophilic asthma, which correlates with neutrophilia and clinical parameters such as PFTs [15,16]. In this context, Besteman et al. observed a strong expression of genes of the NF-κB signalling pathway after RSV infection [17]. Goossens et al. studied sputum samples of patients with asthma and identified a cluster of three asthma endotypes by using sputum transcriptome clustering and dividing them into pauci-granulocytic non-eosinophilic, non-neutrophilic inflammation; eosinophilic inflammation; and neutrophilic inflammation [18]. Further analysis by Fricker et al. demonstrated a pathophysiology-relevant macrophage transcriptome profile in the endotype cluster of neutrophilic asthma that contributes actively to inflammation [19]. These findings underline the important role of the NF-κB pathway in neutrophilic inflammation and demonstrate the active involvement of macrophages in inflammatory lung diseases.

We identified genes, such as *TNFRSF1* (*TNFR1*), *TRAF1*, *CFLAR* (*c-FLIP*), *RELA* (*p65*), *NFKBIA* (*IκBα*), and *BIRC3* (*cIAP2*), that primarily enhance anti-apoptotic and pro-inflammatory processes via the TNF-α/NF-κB signalling pathway [20,21]. Tumour necrosis factor receptor (TNFR)-associated factor 1 (TRAF1) regulates NF-κB signalling and is involved in persistent inflammation. In addition, TRAF1 stabilises cellular apoptosis inhibitor protein 2 (cIAP2) [22]. In this context, Pryhuber et al. found that the *TRAF1* and *cIAP2* genes are highly regulated in lung cells and play an important role in lung inflammation [23]. Together, these gene products form a stable complex I, ubiquitinate RIPK1, which promotes the activation of RELA (p65)/NF-κB and upregulates proinflammatory cytokines (TNF-α, IL-6, IL-8) and other anti-apoptotic factors [24]. NFKBIA acts as a negative feedback inhibitor of NF-κB, whose upregulation indicates intense signalling activity. CFLAR (c-FLIP) and BIRC3 inhibit caspase-8 in apoptotic complex II, thereby blocking TNFR1-induced cell death (apoptosis/necroptosis), allowing cells to survive longer and continue to produce inflammatory mediators [25,26].

In contrast to the increased expression of inflammatory genes, there was a significant downregulation of the respiratory chain genes *MT-ND2*, *NDUFAB1*, *NDUFB7*, *NDUFB8*, *NDUFS3*, *NDUFS5*, *NDUFS7*, and *MT-CO3*, indicating mitochondrial dysfunction in sputum-derived cells from the PiBO patients.

Mitochondria play a key role in inflammatory lung diseases [27]. They regulate apoptosis and biosynthetic metabolic pathways and are essential for inflammatory responses and leukocyte survival [28,29]. With the exception of cytochrome c oxidase (MT-CO3), which is found in complex IV, the identified genes in our study belong to complex I of the respiratory chain. The downregulation of genes encoding these NADH dehydrogenases leads to impairment of the mitochondrial respiratory chain with increased superoxide production and reduced ATP synthesis [30]. ROS activates the NF-κB signalling pathway in a redox-sensitive manner. It inactivates inhibitors (IκBα), activates the IKK complex, and enables NF-κB nuclear translocation, which promotes the transcription of proinflammatory genes. At the same time, transcription NF-κB, together with other transcription factors, activate the SOD2 promoter region, leading to compensatory upregulation [31,32]. These findings were reflected in our study with regard to the reduced expression of cytochrome c (CYCS) and MT-CO3 as well as the upregulation of superoxide dismutase 2 (SOD2). Consequently, the results suggest that the dysregulation of respiratory chain genes is involved in maintaining inflammatory processes.

In addition, chronic inflammatory reactions in the lungs have been shown to involve a transition from oxidative phosphorylation (OXPHOS) of the mitochondria to a higher rate of glycolysis for ATP production in pro-inflammatory immune cells such as inflammatory M1 macrophages and Th1 and Th17 lymphocytes [33]. This transition from OXPHOS to an increased rate of glycolysis, known as the Warburg effect, involves PFK2 (6-phosphofructo-2-kinase/fructose-2,6-bisphosphatase 3) and is activated by hypoxia-inducible factor 1-alpha (Hif-1α). Both play a central role in this transition from oxidative phosphorylation to an increased rate of glycolysis for ATP production [29,33]. Accordingly, the transcription factor Hif-1α as well as PFK2 were found to be overexpressed in the sputum of PiBO patients.

The upregulation of genes in the NF-κB signalling pathway, coupled with the downregulation of genes in mitochondrial complex I/IV (*MT-CO3*, *MT-ND2*, *NDUF* subunits), indicates chronic inflammatory dysfunction of the respiratory tract, in which oxidative stress due to mitochondrial deficits enhances NF-κB-mediated proinflammatory signalling and destabilises the electrochemical gradient, ultimately leading to energy deficiency.

Since our transcriptome analyses do not allow for drawing cell-type-specific conclusions, we performed linear regression analyses to identify which cells are likely to be drivers of these effects. Similar to other chronic inflammatory lung diseases, such as acute respiratory distress syndrome (ARDS), asthma, cystic fibrosis (CF), and chronic obstructive pulmonary disease (COPD), both cell types, neutrophils and macrophages, seem to play key roles in maintaining inflammation in PiBO [34,35]. This is supported by our previous sputum data, which showed that calprotectin, a marker for neutrophilic inflammatory processes, as well as the expression of IL-1b, IL-8, and the macrophage marker MARCO, were significantly dysregulated in the sputum of these patients [36]. Therefore, we used the linear regression analysis to model correlations between the expression of the DEGs and neutrophil and macrophage counts. In addition, we also analysed the influence of the genes on FEV1.

Interestingly, both DEGs of the NF-κB signalling pathway and DEGs of the OXPHOS showed stronger correlations with macrophages than with neutrophils. Together with the mIHC analyses and the in vitro experiments, our data indicate an important role for macrophages in the disease process. As expected, mIHC analysis underlined neutrophilia in the sputum of PiBO patients, but also revealed that there is a trend of reduction in the total number of AMs associated with a shift in macrophage polarisation. This dominance of activated inflammatory M1 macrophages explains their contribution to the maintenance of chronic inflammation [37]. The close relationship between NFKB-related gene expression and macrophage activation is supported by the in vitro experiments that demonstrated a significant increase in RELA and CFLAR expression in macrophages but not in neutrophils.

Furthermore, the reduced AMs numbers also explain the downregulation of respiratory chain gene expression. The polarisation of macrophages into proinflammatory M1 cells is accompanied by metabolic reprogramming, in which OXPHOS is reduced in favour of glycolysis [38,39]. This was also found in our in vitro experiments; M1 macrophages showed lower expression of NUDFB8 and NUDFS5 compared to AMs in the inflammatory setting. Thus, the absence of AMs leads to reduced total expression of respiratory chain genes.

Notably, DEGs of the NFκB signalling pathway and DEGs of the mitochondrial respiratory chain correlated with FEV_1_ lung function, indicating a significant impact of their deregulation on clinical findings. In this regard, alongside macrophages and neutrophils, epithelial cells also play an important role in regulating inflammation in the lungs [34,40]. For this reason, we stimulated alveolar lung cells. We also found genes of the TNFα-dependent NFκB signalling pathway that were upregulated and genes of oxidative phosphorylation that were downregulated.

Although neutrophilic inflammation dominates in PiBO, macrophages seem to play an important role in the regulation of chronic inflammation. AMs regulate the resolution of inflammation in the lungs by being reprogrammed into M2 cells by releasing anti-inflammatory mediators, such as IL-10, TGF-β, and lipid mediators; repairing the epithelium; and preventing excessive damage. Dysfunctions lead to chronic diseases such as COPD [41,42]. Since impaired or incomplete resolution of inflammation is considered to be a central mechanism in the course of the disease, the loss of AMs and/or the polarisation to M1 macrophages could be responsible for the maintenance of chronic inflammation in PiBO.

The question, however, is whether AMs polarise into M1s or whether they are lost and new M1 macrophages are recruited instead. In principle, alveolar macrophages (AMs) can adopt M1-like states of activation. In severe pneumonia, parts of the tissue-specific AMs are replaced by monocyte-derived, proinflammatory macrophages [43,44]. Thus, in PiBO, both scenarios appear plausible: polarisation of local AMs or recruitment of new M1-like macrophages. Studies using methods such as single-cell RNA sequencing are needed to understand the role and importance of macrophage activation and recruitment. If macrophages are the primary drivers of PiBO, this knowledge would enable a variety of new treatment strategies. For example, CCR2 antagonists block the recruitment of monocytes into areas of pulmonary inflammation [45]. New CCL2/CCR2 antagonists are currently being tested for various inflammatory and fibrotic diseases in clinical trials and could potentially be considered as a therapeutic option in PiBO.

To promote the repolarisation of proinflammatory M1 macrophages into anti-inflammatory M2 macrophages, IL-4/IL-13 analogs and PPARγ agonists such as pioglitazone are being used as they preferentially target the M2 phenotype and thereby reduce proinflammatory cytokines (TNF-α, IL-1β) [46,47]. In view of this, inhibiting inflammatory signalling pathways in macrophages could present another promising approach to suppress pro-inflammatory M1 activation.

Our studies show that modulation via transfection with miRNA mimics and inhibitors has a strong anti-inflammatory effect on NF-κB-mediated inflammation [13].

In case of persistent inflammation, macrophages fail to “switch off” the inflammatory response. Pro-resolving mediators such as lipoxins and resolvins promote efferocytosis (the phagocytosis of dead cells), enhance the clearance function, and initiate reprogramming into anti-inflammatory M2 phenotypes, thereby accelerating tissue repair [9,48].

Macrolides (e.g., azithromycin, erythromycin) have been shown to influence macrophage polarisation through immunomodulatory effects in preclinical studies [49,50]. There are reports that Azithromycin, as part of the FAM regime, may have some benefit to PiBO patients [51]. Further studies are needed to investigate the specific efficacy of macrolide treatment in PiBO.

There are limitations within this study. Due to the rarity of the disease, the number of patients is small. This applies in particular to the results of the linear regression analyses, which should be interpreted as exploratory and hypothesis-generating rather than confirmatory. Furthermore, obtaining sputum from children and young adults is challenging as its induction is intensive and the success rate and quality of sputum samples are difficult to guarantee, especially in young patients [52,53]. The non-invasive character of sputum samples reflects real-life data on airway inflammation. However, a selection bias might have been induced as only patients who were able to provide good sputum samples were included for analysis. In view of this, the results may not apply to younger children or patients unable to expectorate.

The patients were studied after the initial insult, and neither the involvement of the causing pathogen nor acute inflammatory processes could be traced. In addition, our analysis did not control for parameters such as treatment, especially glucocorticoids, which can influence the sputum transcriptome [53]. However, as all patients were off medication at the time of the sputum induction, we considered the transcriptome findings to be unrelated to those specific parameters. This does not exclude that in larger cohorts, due to patient heterogeneity, more differences might appear. All of our patients reported having had no passive or active contact with smokers. Although air pollution and other environmental factors were denied, these can influence the sputum transcriptome and may still have had an impact that we cannot entirely rule out.

Also, our data show primarily association, not causation; future studies using macrophage-specific OXPHOS inhibitors or rescue experiments are needed.

Nevertheless, our study is unique in the field of PiBO as data with sputum RNA-Seq and mRNA cytokine levels are scarce for this rare paediatric lung condition.

## 5. Conclusions

Our findings demonstrate upregulated TNF-α/NF-κB signalling and downregulated mitochondrial respiratory chain function in sputum cells derived from patients with PiBO. The loss of AMs and polarisation to M1 indicate a central role for macrophages in the immunopathology of PiBO. A better understanding of disease progression within PiBO patients according to their sputum transcriptome will contribute to improve classification and the selection of appropriate treatment.

## Figures and Tables

**Table 1:** Patient characteristics.

	Controls	Patients	*p*-Value
**Number**	n = 8	n = 9	---
Age (years)	19.9 ± 7.9	16.7 ± 6.9	n.s.
Gender (m/f)	3/5	7/2	---
FVC (%)	97.9 ± 16.0	78.3 ± 12.4	<0.05
FEV1 (%)	99.4 ± 10.6	61.8 ± 15.9	<0.0001
FEV1/FVC	0.87 ± 0.07	0.67 ± 0.10	<0.0001
FEF75 (%)	103.5 ± 20.2	32.4 ± 20.2	<0.0001
Total cells (10^4^/mL)			
Neutrophils (%)	25.6 ± 21.0	73.6 ± 14.8	<0.0001
Macrophages (%)	73.4 ± 20.0	24.5 ± 14.4	<0.0001
Lymphocytes (%)	1.43 ± 1.01	1.47 ± 1.93	n.s.

Values are shown as mean ± standard deviation.

**Table 2:** Linear regression analysis of DEGs in relation to FEV1 neutrophils and macrophages.

Gene	FEV1	Neutrophils	Macrophages
TNFRSF1	*p* < 0.01, R^2^ = 0.38	ns, R^2^ = 0.08	*p* < 0.01, R^2^ = 0.52
TRAF1	*p* < 0.05, R^2^ = 0.35	ns, R^2^ = 0.14	ns, R^2^ = 0.20
CFLAR	*p* < 0.01, R^2^ = 0.37	ns, R^2^ = 0.19	*p* < 0.01, R^2^ = 0.45
NFkB1A	ns, R^2^ = 0.08	ns, R^2^ = 0.02	*p* < 0.05, R^2^ = 0.28
RELA	*p* < 0.01, R^2^ = 0.49	*p* < 0.05, R^2^ = 0.25	*p* < 0.05, R^2^ = 0.28
BIRC3	ns, R^2^ = 0.23	ns, R^2^ = 0.17	ns, R^2^ = 0.20
MT-CO3	*p* < 0.05, R^2^ = 0.36	*p* < 0.05, R^2^ = 0.34	*p* < 0.01, R^2^ = 0.44
MT-ND2	*p* < 0.01, R^2^ = 0.52	*p* < 0.05, R^2^ = 0.29	*p* < 0.01, R^2^ = 0.54
NDUFAB1	*p* < 0.01, R^2^ = 0.38	*p* < 0.01, R^2^ = 0.43	*p* < 0.001, R^2^ = 0.60
NDUFB7	ns, R^2^ = 0.22	ns, R^2^ = 0.23	*p* < 0.05, R^2^ = 0.37
NDUFB8	*p* < 0.01, R^2^ = 0.42	*p* < 0.01, R^2^ = 0.54	*p* < 0.0001, R^2^ = 0.75
NDUFS3	*p* < 0.01, R^2^ = 0.40	ns, R^2^ = 0.08	*p* < 0.01, R^2^ = 0.40
NDUFS5	*p* < 0.01, R^2^ = 0.40	*p* < 0.05, R^2^ = 0.30	*p* < 0.0001, R^2^ = 0.73
NDUFS7	*p* < 0.01, R^2^ = 0.52	ns, R^2^ = 0.23	*p* < 0.001, R^2^ = 0.63

Values are shown as *p*-value and coefficient of determination.

## Data Availability

The MACE-seq analysis data are deposited in GEO and are scheduled for public release on 30 June 2026, under accession number GSE327957.

## Supplementary Materials

The following supporting information can be downloaded at https://www.mdpi.com/article/10.3390/biom16050745/s1, Figure S1: Correlations between the identified target genes and FEV1, neutrophils, and macrophages.

## Abbreviations

BALF	Bronchoalveolar lavage fluid
DEGs	Differentially expressed genes
HRCT	High-resolution computed tomography
mIHC	Multiplex immunohistochemistry
OXPHOS	Oxidative phosphorylation
PFT	Pulmonary lung function
PiBO	Post-infectious bronchiolitis obliterans
R^2^	Coefficient of determination
