# Supplementary material for: Transcriptome Profiling of Induced Sputum Identified Upregulated TNF-α/NF-κB Signalling and Downregulated Mitochondrial Respiratory Chain Function in Post-Infectious Bronchiolitis Obliterans
Source: Biomolecules. 2026 May 19;16(5):745. doi: 10.3390/biom16050745 (PMC13204173; doi:10.3390/biom16050745)
Supplement: Supplementary file 1 [file biomolecules-16-00745-s001.zip › biomolecules-4294286-supplementary.pdf]

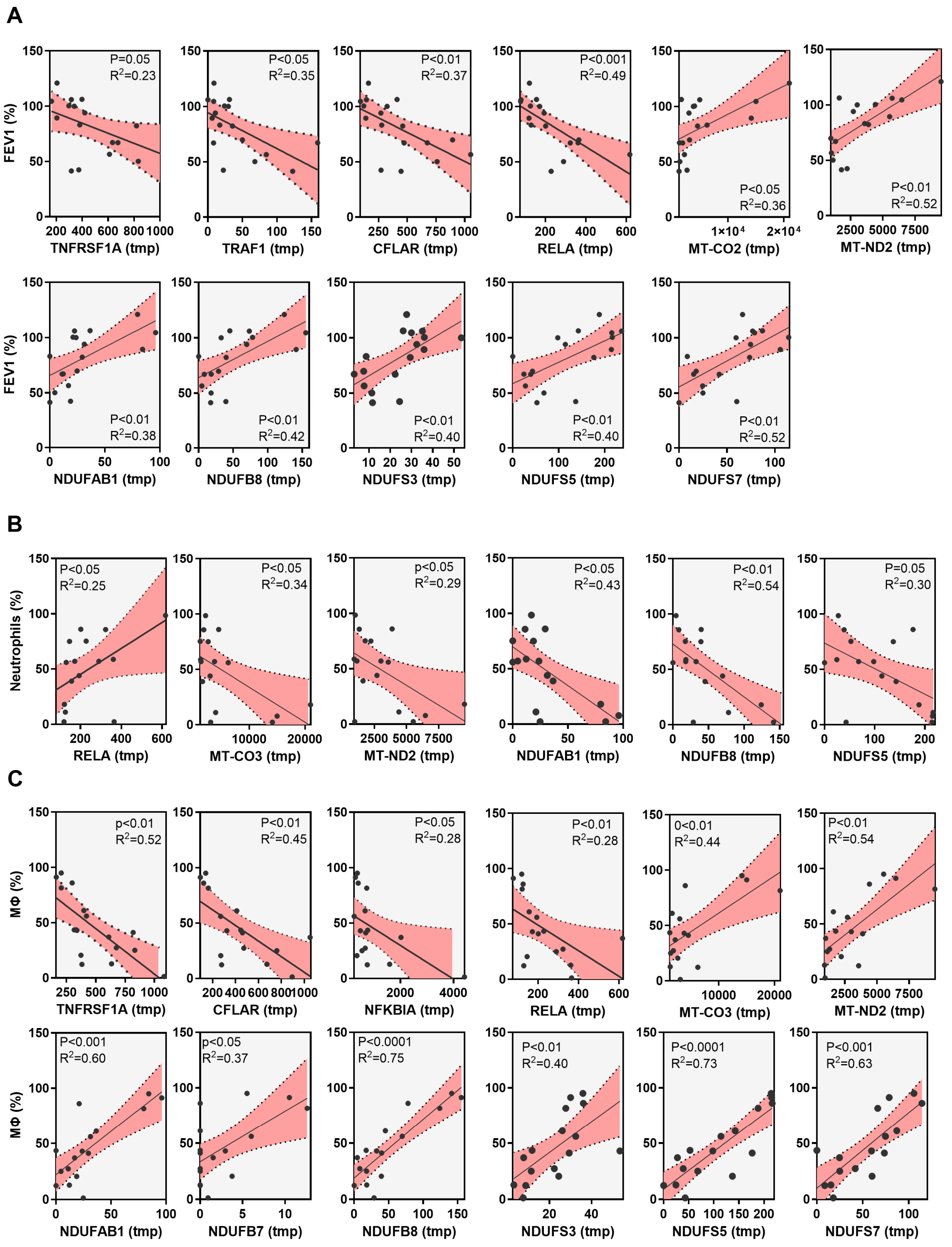

Supplementary Figure S1: Correlations between the identified target genes and FEV1, neutrophils, and macrophages. Linear regression was performed to model correlations between the expression of the DEGs and **(A)** FEV1 lung function, **(B)** neutrophils and **(C)** macrophages count. The graphs show a comparison of DEG expression with FEV1 values, as well as neutrophil and macrophage counts. Each graph displays the regression line with a 95% confidence interval (shaded area). The coefficient of determination (R^2) and the p-value are provided.
